# Interactions Between the Ubiquitin–Proteasome System, Nrf2, and the Cannabinoidome as Protective Strategies to Combat Neurodegeneration: Review on Experimental Evidence

**DOI:** 10.1007/s12640-024-00694-3

**Published:** 2024-02-23

**Authors:** Luis Angel Monsalvo-Maraver, Enid A. Ovalle-Noguez, Jade Nava-Osorio, Marisol Maya-López, Edgar Rangel-López, Isaac Túnez, Alexey A. Tinkov, Yousef Tizabi, Michael Aschner, Abel Santamaría, Students from Programa Delfín 2022, Diana C. Medrano-Cruz, Uriel García-Flores, Sara I. Castañeda-Roque, Dan L. Romero-Méndez, Elizabeth Varillas-Galicia, Daniela Rojas-Lobato, Santiago Navas-Escobar, Karen Rentería-Pérez

**Affiliations:** 1 Facultad de Ciencias, Universidad Nacional Autónoma de México, Av. Universidad 3000, C.U. Coyoacán, 04510 Mexico City, Mexico; 2 Doctorado en Ciencias Biológicas y de La Salud, Universidad Autónoma Metropolitana-Iztapalapa, Mexico City, Mexico; 3 Instituto Nacional de Neurología y Neurocirugía, S.S.A., Mexico City, Mexico; 4 Instituto de Investigaciones Biomédicas Maimonides de Córdoba (IMIBIC), Departamento de Bioquímica y Biología Molecular, Facultad de Medicina y Enfermería, Universidad de Córdoba, Red Española de Excelencia en Estimulación Cerebral (REDESTIM), Córdoba, Spain; 5 IM Sechenov First Moscow State Medical University (Sechenov University), Moscow, Russia; 6 Yaroslavl State University, Yaroslavl, Russia; 7 Department of Pharmacology, Howard University College of Medicine, Washington, DC USA; 8 Department of Molecular Pharmacology, Albert Einstein College of Medicine, Bronx, NY USA

**Keywords:** System interaction, Endocannabinoidome, Ubiquitin–proteasome system, Nrf2/Keap-1/ARE system, Neuroprotection, Neurodegeneration

## Abstract

Neurodegenerative disorders are chronic brain diseases that affect humans worldwide. Although many different factors are thought to be involved in the pathogenesis of these disorders, alterations in several key elements such as the ubiquitin–proteasome system (UPS), the nuclear factor erythroid 2-related factor 2 (Nrf2) signaling pathway, and the endocannabinoid system (ECS or endocannabinoidome) have been implicated in their etiology. Impairment of these elements has been linked to the origin and progression of neurodegenerative disorders, while their potentiation is thought to promote neuronal survival and overall neuroprotection, as proved with several experimental models. These key neuroprotective pathways can interact and indirectly activate each other. In this review, we summarize the neuroprotective potential of the UPS, ECS, and Nrf2 signaling, both separately and combined, pinpointing their role as a potential therapeutic approach against several hallmarks of neurodegeneration.

**Graphical Abstract:**

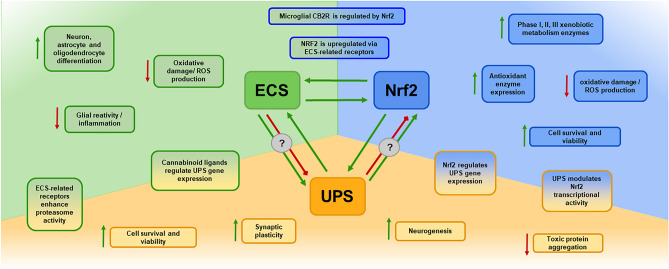

## Introduction

Neurodegenerative disorders (NDs) include a group of chronic brain diseases characterized by progressive neuronal death within specific regions of the nervous system. Human pathologies like Alzheimer’s disease (AD), Parkinson’s disease (PD), Huntington’s disease (HD), and amyotrophic lateral sclerosis (ALS) are included in this group and currently represent a significant public health problem (Jellinger 2003; Fu et al. 2018).

In most cases, the origin of NDs is not fully understood, and it is commonly associated with a complex mixture of genetic and environmental factors (Coppede et al. 2006). Phenomena such as overproduction of reactive oxygen species (ROS) lead to oxidative stress (OS), mitochondrial dysfunction and neuroinflammation (Picca et al. 2020), as well as endoplasmic reticulum stress (ERS) (Xiang et al. 2017), and excitotoxic damage (Doble 1999), which are all linked to NDs’ origin and progression.

One of the main causal factors of NDs is related to misfolded proteins which accumulate within cells, triggering toxic mechanisms that disturb neuronal homeostasis and ultimately provoke cell death. Proteins such as amyloid-β (Aβ, related to AD), α-synuclein (α-Syn, related to PD), huntingtin (Htt, related to HD), and tau (related to AD and other tauopathies) have emerged as potential culprits regarding neurodegeneration. While it is true that the cause of the accumulation and toxic effects of these proteins remains unclear, increasing evidence suggests that an impairment in the mechanisms responsible for maintaining cellular proteostasis, such as the autophagosome-lysosome system and the ubiquitin–proteasome system (UPS), might allow these proteins to aggregate and exert toxicity on neural cells (Takalo et al. 2013; Zheng et al. 2016; Di Meco et al. 2020; Monsalvo-Maraver et al. 2023).

Although many experimental therapeutic approaches have been developed to treat NDs, targeting cannabinoid signaling and redox-related pathways, such as Nrf2, has shown promising results against oxidative stress, neuroinflammation, and other related toxic mechanisms that compromise cell function (van Muiswinkel and Kuiperij 2005; Fagan and Campbell 2014). This review is aimed to describe the interactions between key systems involved in the maintenance of neural cell homeostasis, specifically (1) the ubiquitin–proteasome system (UPS), (2) the endocannabinoidome, and (3) the Nrf2 signaling pathway, also discussing the role that these interactions might play in NDs’ pathology and potential treatment.

## Ubiquitin–Proteasome System (UPS)

### Overview

In eukaryotes, the proteasome is the major protein-degrading complex within the cytosolic and nuclear compartments (Bard et al. 2018). Structurally, the eukaryotic 26S proteasome is composed of two subcomplexes: the 20S core particle and the 19S regulatory particle (Schweitzer et al. 2016). The core particle contains the proteolytic fraction of the proteasome and it is formed by four heptameric rings organized in two pairs: two outer α-rings and two inner β-rings that together form a hollow chamber, in which protein substrates are degraded (Groll et al. 1997; Kopp et al. 1997). In turn, the 19S particle corresponds to the regulatory fraction responsible for recognizing, displaying, translocating, and deubiquitinating substrates for the core particle (Budenholzer et al. 2017).

The proteasome targeted proteins are identified by cells using ubiquitin, a small protein that is attached to lysine residues of other proteins within the cell, thus modifying their function, leading them to interact with other molecules, or altering their localization within the cell compartments; however, ubiquitin is also attached to proteins targeted for proteasomal degradation through a process that requires ubiquitin-activating enzymes (E1), ubiquitin-conjugating enzymes (E2), and ubiquitin ligases (E3). Given that the 19S subcomplex recognizes ubiquitin-attached proteins, allowing them to enter the 20S core for degradation, the ubiquitination machinery and the 26S proteasome form together a whole proteolytic pathway called the UPS (Komander and Rape 2012; Ehlinger and Walters 2013; Kleiger and Mayor 2014; Watanabe et al. 2020).

### Physiological Function of UPS in the Nervous System

Within the nervous system, UPS has been reported to be involved in a number of cell processes. Firstly, as early as the embryonic neural tube is being formed, the UPS-ubiquitin ligase protein F-box protein 30 (FBXO-30) mediates the degradation of the retinoic acid receptor, being retinoic acid a vital molecule for neural tube formation (Cheng et al. 2019). Furthermore, UPS appears to modulate differentiation of several cell types of the nervous system as evidence suggests that (1) in *Drosophila melanogaster*, E3 proteins Slimb and Ago allow glial progenitors to stop proliferating and differentiate into specific lineages (Ho et al. 2009); (2) UPS is involved in motor neuron differentiation, and ubiquitin-like modifier activating enzyme 1 E1 (UBA1) inhibition results in decreased motor neuron viability and neurite growth (Bax et al. 2019); (3) mRNA levels of several proteasome components within the mesencephalon change during postnatal development in rats, suggesting that UPS regulates neuronal development of structures such as the *Substantia nigra pars compacta* (El-Khodor et al. 2001); and (4) proteasome inhibition appears to be necessary for oligodendrocyte differentiation (Pasquini et al. 2003).

UPS components have also been shown to be necessary for axon growth and guidance during development in different animal models. Regarding axon growth, Highwire E3 protein has been reported to be related to neuromuscular synapse formation in *D. melanogaster* (DiAntonio et al. 2001), whereas in *Xenopus*, neural precursor cell expressed developmentally down-regulated protein 4 (Nedd4; an E3-enzyme) proved to enhance axon branching in retinal ganglion cells (Drinjakovic et al. 2010). In mammals, another E3-enzyme (anaphase-promoting complex or APC) mediated axon growth as shown in rat cerebellar granule neurons (Konishi et al. 2004). On the other hand, E3-enzymes Highwire, RPM-1, and Phr1 are strongly related to axon guidance regulation as studies have shown that (1) Highwire loss prevents sister axons from diverging to target the two lobes of the mushroom body; an important structure for learning in *Drosophila* sp. brain (Shin and DiAntonio 2011); (2) RPM-1 mutants display axon guidance disturbances within mechanosensory neurons of *Caenorhabditis elegans* (Schaefer et al. 2000); and (3) mice with Phr-1 deficiency exhibit motor neurons with an altered axon guidance in the spinal cord, while axonal growth cones and axons present aberrant morphologies (Lewcock et al. 2007).

Synaptic plasticity is another phenomenon closely related to UPS function. Firstly, studies have reported that Calmodulin-kinase II (CaMKII) enhances proteasome migration towards dendritic spines upon glutamatergic receptor-dependent synaptic stimulation (Bingol and Schuman 2006), and after proteasome migration, CaMKII is able to phosphorylate Rpt-6 proteasome subunit, consequently enhancing dendritic spine growth as shown in hippocampal slice cultures (Bingol et al. 2010; Hamilton et al. 2012). Moreover, UPS is also thought to take part in long-term potentiation (LTP), as evidence suggests that proteasome inhibition upregulates early-phase LTP and facilitates induction of late-phase LTP; however, it also disrupts late-phase LTP maintenance and stability. Noteworthy, other studies show that UPS is necessary for the induction, but not the maintenance, of late-phase LTP, suggesting that available evidence still remains controversial (Fonseca et al. 2006; Dong et al. 2008; Cai et al. 2010; Santos et al. 2015).

It has also been proposed that UPS plays a major role during neurogenesis, as proteasome-assembly chaperone Gankyrin was shown to enhance neurogenesis in neural stem cells and UPS activity inhibition decreases neural stem cell proliferation (Sahu et al. 2019; Singh et al. 2023). Combined, the evidence above described supports the concept that UPS is involved in several processes regarding physiological functions in the nervous system.

### Role in Physiopathology of Neurodegenerative Disorders

Since the vast majority of NDs are closely related to abnormally aggregated proteins and their cytotoxic effects, UPS has emerged as a potential therapeutic target to antagonize and inhibit protein-related damage. When the proteasome is experimentally inhibited, aberrant accumulation of Aβ, α-Syn, phosphorylated-Tau (p-Tau), and Htt is exacerbated, as shown with different cell culture models (Zhou et al. 2003; Casarejos et al. 2011; Agholme et al. 2012); notably, proteasome activity reduction was observed in purified proteasomes exposed to Aβ-oligomers (Tseng et al. 2008) and filamentous Htt (Díaz-Hernández et al. 2006), as well as in PC12 cells exposed to mutant α-Syn, and in cultured neurons overexpressing human Tau (Tanaka et al. 2001; Ye et al. 2020).

In contrast, it has been reported that proteasome activity potentiation is able to diminish the toxic effects of ND-related proteins. For instance, (1) the triterpenoid ursolic acid has been used to activate the proteasome, thereby lessening paralysis in *C. elegans* nematodes exposed to Aβ (Wang et al. 2022a, b, c); (2) *Gastrodia elata* extracts can activate the proteasome, thus protecting PC12 cells from cell viability reduction induced by Htt (Huang et al. 2011); (3) inducing UPS-mediated degradation of Tau in N2a cells restored normal mitochondrial distribution within cells and rescued viability of Aβ-treated cells (Chu et al. 2016). Combined, this evidence suggests that UPS activity enhancement could be used to target proteostasis impairments in NDs. In addition, other hallmarks of the physiopathology of NDs could be reproduced under experimental conditions by proteasome inhibition. Glial reactivity and motor alterations were reported after an in vivo nigral injection of lactacystin (a proteasome inhibitor) in mice (Deneyer et al. 2019). In several cell culture models, proteasome inhibitors have been shown to reduce neuronal survival and viability, while enhancing ROS production, caspase activity, proapoptotic protein expression and mitochondrial translocation, mitochondrial membrane depolarization, and apoptosis-inducing factor (AIF) and cytochrome c release to the cytosol. Furthermore, UPS inhibitors also decreased the levels of antiapoptotic proteins, diminished (GSH) and oxidized (GSSG) glutathione levels, as well as synapse-related protein levels of βIII-tubulin, drebrin, and synaptophysin (Kikuchi et al. 2003; Papa et al. 2007; Bajic et al. 2012).

A significant amount of evidence is available on the neuroprotective actions derived from UPS upregulation. Table 1 summarizes some of the most relevant reports on this topic.

**Table 1:** Main experimental results of studies reporting neuroprotective effects of UPS stimulation against NDs: AD, PD, HD, and ALS. *18α-GA* 18α-glycyrrhetinic acid; *Akt* protein kinase B; *DBTO* (1R,3E,6R,7Z,11S,12S)-dolabella-3,7,18-trien-6,17-olide; *GABA*_*B*_ γ-aminobutyric acid receptor B; *LMP7* 20S proteasome subunit β 8; *MPP* + 1-methyl-4-phenylpyridinium; *mTOR* mechanistic target of rapamycin; *PAP1* proteasome-activating peptide 1; *PA28γ* proteasome activator 28γ; *PKA* protein kinase A; *p60S6K* p60S6-kinase; *PSMD1* 26S proteasome regulatory subunit N2

Disease	Experimental approach	Main results of UPS potentiation	Reference
AD	5xFAD mice (transgenic AD mice model) were treated with a combination of 18α-GA and omega-3 fatty acids (proteasome activators)	Proteasome activator exposure improved proteasome activity while reducing Aβ coverage within both the hippocampus and cortex of 5xFAD mice. In vivo, this treatment also enhanced vertical and locomotor activity of the rodents	(Djordjevic et al. 2021)
	SH-SY5Y cells were exposed to Aβ toxicity and treated with DBTO (proteasome activator)	DBTO treatment successfully enhanced proteasome activity in SH-SY5Y cultures, consequently improving cell survival to Aβ cytotoxic effects	(Vasilopoulou et al. 2022)
	3x-Tg transgenic mice were fed for 10 months with a diet supplemented with resveratrol	Resveratrol induced an increase in proteasome activity and protein levels of 20S core particles, which was accompanied with a reduction of ubiquitinated proteins, phosphorylated Tau, and Aβ oligomers	(Corpas et al. 2019)
	SH-SY5Y cell cultures were transfected to express truncated forms of Tau protein. Afterwards, it was evaluated the neuroprotective potential of geldanamycin in the cultures	The expression of the truncated form of Tau protein led to the generation of intracellular aggregates and the inhibition of the activity of proteasome. Additionally, pharmacological inhibition of proteasome resulted in an increased aggregation of Tau. Conversely, geldanamycin treatment enhanced the proteasome activity, consequently augmenting tau degradation	(Opattova et al. 2013)
	Several *C. elegans* strains were used to evaluate the neuroprotective potential of 18α-GA (proteasome activator) against Aβ-induced toxicity	18α-GA treatment proved to increase the lifespan of *C. elegans* in a proteasome-dependent manner. Furthermore, 18α-GA decreased Aβ accumulation and Aβ-dependent paralysis in the nematodes	(Papaevgeniou et al. 2016)
PD	SH-SY5Y cell cultures were exposed to MPP + toxicity. Subsequently, the cells were treated with puerarin (isoflavone isolated from *Pueraria lobata*)	Puerarin improved proteasome function in the cultured cells. In this study, an increase in cell viability and a reduction in apoptotic death markers in response to puerarin treatment were reported	(Cheng et al. 2009)
	Primary neuron cultures were obtained from M83 mice (which express human α-Syn). PC12 and N2a cells (transfected with a α-Syn-expressing plasmid) were also used. All cultures were treated with harmine	Harmine proved to increase α-Syn clearance in all cell models. This degradation was UPS-dependent. Additionally, harmine was found to act through PKA to induce the expression of PSMD1, a subunit of the 19 s regulatory particle of proteasome, consequently enhancing proteasome activity	(Cai et al. 2019)
	PC12-derived cell lines overexpressing α-Syn were cultured and exposed to a tetramethylpyrazine derivative (T-006). Mice overexpressing α-Syn were also used for assessing the neuroprotective potential of T-006	In pC12 cells, T-006 acted though the PKA/Akt/mTOR/p60S6K to increase LMP7 expression. LMP7 expression enhanced overall proteasome activity, thereby promoting UPS-dependent α-Syn degradation In α-Syn-expressing mice, T-006 reduced dopaminergic neuronal loss and improved the in vivo performance on the rotarod test	(Zhou et al. 2019)
	SH-SY5Y neuroblastoma cell cultures were exposed to MPP + toxicity. Afterwards, demethylsuberosin (isolated from *Cudrania tricuspidata*) was tested as a neuroprotective approach	It was reported an increase in chymotrypsin-like, trypsin-like, and caspase-like proteasome activity in SH-SY5Y cultures after demethylsuberosin treatment. These effects were accompanied by a reduction of the cell death induced by MPP + toxicity	(Kim et al. 2015)
HD	Striatal neurons expressing mutant Htt were transfected with the gene PA28γ (proteasome activator)	PA28γ transfection enhanced the proteolytic activity of the proteasome while diminishing the production of huntingtin in striatal neurons	(Seo et al. 2007)
	YAC128 mice (HD model) were treated with an intrastriatal injection of a lentiviral vector containing the gene PA28γ (proteasome activator)	Mice overexpressing PA28γ proved to enhance proteasome activity and BDNF expression, while reducing ubiquitin-positive inclusion bodies within the striatum, and motor alterations as assessed with rotarod test	(Jeon et al. 2016)
	(1) Striatal neuron-derived cell lines (CD43: expressing mutant Htt and CD19: expressing wild-type Htt) and (2) mutant Htt-expressing YAC128 transgenic mice were used to test neuroprotective effects of Baclofen: a GABA_B_ receptor agonist	In the CD43 cell line, Baclofen exposure increased the cell viability while enhancing the activity of the proteasome. Withstanding the above, in YAC128 mice, Baclofen augmented the proteasome activity and the expression of the β subunit of the 20 s particle. Baclofen also improved rotarod test performance of the mice	(Kim and Seo 2014)
ALS	SH-SY5Y cells were transfected with a mutant version of SOD1 gene carrying the G93A substitution. Afterwards, SH-SY5Y^G93A^ cultures were treated with PAP1, a proteasome activator	PAP1 treatment decreased the aggregation of mutant SOD1 within SH-SY5Y^G93A^ cells. PAP1 also reduced the production of oxidized proteins in SH-SY5Y^G93A^ cells exposed to H_2_O_2_	(Dal Vechio et al. 2014)

## The Endocannabinoid System (ECS)

### Overview

The endocannabinoid system (ECS) is a complex signaling pathway that involves cannabinoid receptors, their endogenous ligands, and the enzymes responsible for ligand biosynthesis and degradation (Lu and Mackie 2021). There are two main ligands belonging to the ECS: anandamide (AEA) and 2-arachidonoyl-glicerol (2-AG), both of which are usually termed endocannabinoids. AEA and 2-AG belong to N-acylethanolamine and 2-acylglycerol families, respectively, and several other molecules from these families share target receptors and metabolic pathways with endocannabinoids, such as oleoylethanolamide and linoleoylethanolamide (N-acylethanolamines), or 2-oleoyl-glycerol and 2-linoleoyl-glycerol (2-acylglycerols). Moreover, other similar molecules like lipoamino acids (N-acyl-serines or N-acyl-taurines) and N-acyl neurotransmitters (N-acyl-dopamines or N-acyl-serotonins) are able to share catabolic enzymes and target receptors with AEA and 2-AG (Cristino et al. 2020).

The two main endocannabinoid receptors are termed cannabinoid receptors 1 (CB1R) and 2 (CB2R), which are conceived as G protein–coupled receptors (GPCR) that signal through Gα_i/o_ G proteins (Hua et al. 2016). Both CB1R and CB2R share a similar tridimensional structure, although they have certain structural differences within their binding sites for cannabinoids, which make them possess different affinities for distinct ligands (Li et al. 2019a, b).

In addition to CB1R/CB2R receptors, further evidence has suggested that endocannabinoids and other related ligands can signal through a number of receptors. Transient receptor potential ion channels of vanilloid type 1–4 (TRPV1-4), transient receptor potential ion channel of melastatin type 8 (TRPM8), transient receptor potential ion channel of ankyrin type 1 (TRPA1) (De Petrocellis et al. 2007, 2012; Raboune et al. 2014; Redmond et al. 2014), T-type calcium channels Ca_v_3.1–3.3 (Ross et al. 2009), peroxisome proliferator-activated receptors α/γ (PPARα/γ) (O'Sullivan 2016), and G protein–coupled receptors GPR18, GPR55, GPR110, and GPR119 (Kohno et al. 2006; Overton et al. 2006; Sharir et al. 2012; Cristino et al. 2020) have all been shown to be modulated by these ligands.

Endocannabinoids and related ligands utilize various anabolic and catabolic pathways, and some of them share key enzymes for their metabolism. The N-acylethanolamine family of cannabinoids is synthesized via N-acylphosphatidylethanolamine-specific phospholipase d-like hydrolase (NAPE-PLD), while diacyl-glycerol lipase (DAGL) synthesizes 2-acylglycerols. In contrast, fatty acid amide hydrolase (FAAH) and monoacylglycerol lipase (MAGL) degrade N-acylethanolamines and 2-acylglycerols, respectively (Basavarajappa 2007; Lu and Mackie 2021). Furthermore, in addition to their basic metabolism, endocannabinoids can be substrates of cyclooxygenase 2 (COX2), lipoxygenase (LOX), and cytochromes P450, which can catalyze the formation of a number of active cannabinoid-derived metabolites (Rouzer and Marnett 2011).

### Physiological Significance in the Nervous System

The ECS is critical for the normal function and development of the nervous system. CB1R-expressing cells have been detected in the neural folds, brain primordia, and neural tube of chick embryos. Accordingly, 2-AG and AEA ligands, as well as NAPE-PLD, MAGL, FAAH, and DAGL enzymes, were detected in early chick embryos, pointing at the existence of a functional ECS within the first embryonic developmental stages (Psychoyos et al. 2012).

Nervous system cell differentiation and neural circuitry-building are also partially regulated by the ECS. CB1R-dependent signaling has proven to enhance the postnatal and adult-stage differentiation of neural progenitor cells into astrocytes (Aguado et al. 2006), the fasciculation of corticothalamic and thalamocortical axonal tracts in mice (Wu et al. 2010), the differentiation of neocortex-deep layer pyramidal neurons (Paraíso-Luna et al. 2020), and the radial migration of pyramidal neurons (with AEA and 2-AG acting like chemoattractant agents for cell migration) (Díaz-Alonso et al. 2017).

Several cannabinoid receptors regulate neurogenesis within the adult brain. CB1R and TRPV1 are thought to promote neuronal differentiation in the subventricular zone and the dentate gyrus (Jin et al. 2004). Additionally, other studies in cell cultures showed that dentate gyrus-cell proliferation needs co-stimulation of CB1R and CB2R, while proliferation in the subventricular zone is either stimulated by CB1R or CB2R, separately (Rodrigues et al. 2017). Other cannabinoid receptors that stimulate hippocampal neurogenesis are GPR55 (Hill et al. 2018), PPARγ (Esposito et al. 2011), and Ca_v_3.1 (Yabuki et al. 2021).

Another main function of endocannabinoids within the nervous system involves glutamatergic synapse-retrograde regulation. Endocannabinoids are produced in postsynaptic terminals in response to glutamatergic stimulation, acting like a retrograde messenger that inhibits presynaptic release of glutamate by activating rectifying potassium channels and inhibiting N-type calcium channels (Maejima et al. 2001; Guo and Ikeda 2004).

Beyond the cellular level, ECS receptor signaling mediates complex behaviors, as it has been shown in in vivo rodent models. CB1R is expressed in the hypothalamus, and when it is deleted, animals gain less weight and increase their energy expenditure, revealing the role of CB1R on energy balance (Cardinal et al. 2012). Moreover, CB1R is also expressed in the basolateral amygdala, where it appears to modulate the formation of associative fear memories (Tan et al. 2011). Together, this supports the concept that the physiological role of ECS is crucial for the normal function of the nervous system, and consequently, ECS-function alterations could play important roles in the pathogenesis of neurological diseases, including NDs.

### Role in the Physiopathology of Neurodegenerative Disorders

The ECS has emerged as a key factor involved in NDs, both because of its potential as a therapeutic target and its role in ND physiopathology. ECS signaling appears to be disturbed as changes in the expression levels of its components have been reported by several studies. In the hippocampus and entorhinal cortex of *postmortem* AD brains, CB2R and FAAH were overexpressed in glial cells surrounding Aβ aggregates (Benito et al. 2003). Notably, several studies report CB1R levels unchanged in AD *postmortem* brains, while others showed a markedly lowered expression, thus demonstrating that the available evidence still remains controversial (Ramírez et al. 2005; Lee et al. 2010). Other reports have shown that, in PD *postmortem* brains, CB1R levels were increased in the putamen and unchanged in the *Substantia nigra* (SN) when matched to control brains. In addition, CB2R expression was enhanced in the SN and decreased in the putamen, while MAGL levels were decreased in the SN and increased in the putamen (Navarrete et al. 2018). In contrast, HD patients showed a decrease in CB1R activity in several gray matter regions, and that reduction was inversely correlated with the length of the polyglutamine coding region of the Htt gene (Van Laere et al. 2010). Moreover, a CB1R level decrease was also observed in interneurons of the caudate nucleus of HD patients (Horne et al. 2013).

Although ECS alterations in patients do not exhibit a common pattern, experimental potentiation of cannabinoid signaling has shown common promising results as a neuroprotective approach. Notwithstanding the above, several studies suggest that cannabinoid potentiation, particularly through MAGL-dependent 2-AG degradation, could exert toxic effects via the production of COX2-catalyzed 2-AG-derived metabolites (Valdeolivas et al. 2013). Nonetheless, the vast majority of experimental approaches support the neuroprotective properties of ECS enhancement. In this regard, in a considerable number of studies, cannabinoid signaling potentiation depends on cannabinoid receptors to exert its neuroprotective properties, and neuroprotection tends to involve the inhibition of cell apoptosis/increase of cell viability and reduction of proinflammatory mediators and microglial/astroglial reactivity (Table 2). Altogether, this evidence suggests that ECS might target specific processes within the nervous system to antagonize neural degeneration in multiple NDs.

**Table 2:** Main experimental results of studies reporting neuroprotective effects of cannabinoid signaling stimulation against NDs: AD, PD, HD, and ALS. *AMPA* α-amino-3-hydroxy-5-methyl-4-isoxazolepropionic acid receptor; *6-OHDA* 6-hydroxydopamine; *JNK* c-Jun N-terminal kinase; *IL-1β* interleukin 1β; *TNFα* tumor necrosis factor α; *NFkB* nuclear factor-kB; *BEOV* bis(ethylmaltolate) oxidovanadium; *iNOS* inducible nitric oxide synthase; *MPTP* 1-methyl-4-phenyl-1,2,3,6-tetrahydropyridine; *GFAP* glial fibrillary acidic protein; *DAM* disease-associated microglia; *3-NP* 3-nitropropionic acid

Disease	Experimental approach	Main results of cannabinoid signaling potentiation	Reference
AD	In vivo TRPV1 upregulation in APP23/PS45 mice via adenoviral vector injection	TRPV1 upregulation inhibited Aβ production and AMPAR endocytosis, while alleviating hippocampal LTP and spatial learning impairments	(Du et al. 2020)
	P301s (Tau transgenic model) mice treated with the MAGL inhibitor JZL184	MAGL inhibition proved to lessen the levels of different forms of p-Tau, astroglial/microglial reactivity, IL-1β/TNFα production, phosphorylated NF-kB, and cleaved caspase 3, while enhancing PPARγ phosphorylation, expression of antiapoptotic proteins, and cognitive function in the transgenic mice	(Hashem et al. 2021)
	(1) BV2 microglial cell line treated with BEOV (PPARγ activator) and stimulated with Aβ afterwards (2) APPswe/PS1E9 mice treated with BEOV	In both models (1) and (2): BEOV reduced proinflammatory cytokine levels and expression of iNOS/COX2 and inhibited NF-kB pathway. In (2) BEOV decreased astroglial/microglial activation	(He et al. 2021)
	Aβ (1–42) was injected into the hippocampus of male Wistar rats, which were also treated with cannabinoid agonist WIN 55,212–2	WIN 55,212–2 ameliorated Aβ effects through a decrease in hippocampal TNFα levels, NF-kB nuclear translocation, and caspase 3 expression induced by the peptide. In addition, WIN 55,212–2 improved spatial memory of Aβ-treated rats. These effects were mediated by CB1R, CB2R, and PPARγ	(Fakhfouri et al. 2012)
	5xFAD mice were crossed with mice lacking FAAH to obtain double transgenic 5xFAD/FAAH^−/−^ mice (bearing both AD traits and lacking FAAH enzyme)	FAAH deletion in 5xFAD/FAAH^−/−^ mice diminished basal hippocampal glutamatergic transmission alterations observed in 5xFAD mice; double transgenic mice also exhibited improved hippocampal LTP and dendritic spine density of CA1 pyramidal neurons and increased microglial activation with a partial DAM signature	(Ruiz-Pérez et al. 2021)
PD	AEA pre-treated PC12 cell cultures exposed to 6-OHDA toxicity	AEA pretreatment limited 6-OHDA-dependent cell apoptosis via inhibition of the JNK pathway, a result that was independent of CB1/2R and TRPV1 receptors	(Mnich et al. 2010)
	C57BL6 mice were injected with WIN 55,212–2 and HU210 (cannabinoid receptor agonists) before the exposure to MPTP dopaminergic neuron toxin	Cannabinoid agonists, via CB1R activation, inhibited dopaminergic neuronal loss, proinflammatory cytokine production, microglial reactivity, NADPH oxidase-dependent ROS production, oxidative damage to the SN, and alleviated motor behavior deficits in mice in vivo	(Chung et al. 2011)
	Rotenone toxin was injected into Wistar rats, which were also treated with β-caryophyllene (CB2R agonist)	CB2R activation protected the SN from dopaminergic neuronal death, while reducing astroglial/microglial activation, oxidative damage marker-detection, proinflammatory cytokine generation, and levels of inflammation-associated proteins (NF-kB, COX-2, and iNOS)	(Javed et al. 2016)
	C57BL/6 J mice were exposed to a 6-OHDA intrastriatal injection prior to the treatment with GW842166x (CB2R agonist)	CB2R agonist inhibited 6-OHDA-associated damage by diminishing dopaminergic neuron loss and improving the motor deficits observed in 6-OHDA treated mice as tested with rotarod test, pole test, balance beam test, and grip strength test	(Yu et al. 2021)
	C57BL/6 J mice were treated with 2-AG, MAGL inhibitors JZL184/URB602, and/or COX-2 inhibitor DFU. Afterwards, mice were injected intraperitoneally with MPTP toxin	Treatment with either MAGL inhibitors or 2-AG ameliorated dopaminergic cell death induced by MPTP in the SN. However, when JZL184 was co-administered with DFU, dopaminergic cell death reduction was enhanced	(Mounsey et al. 2015)
HD	CB2R agonist HU-308 was administered to mice with a previous intrastriatal injection of quinolinic acid (excitotoxin)	HU-308 treatment lowered striatal medium spiny neuron loss, which was accompanied by lessened motor alterations, brain edema, glial reactivity, and NO production	(Palazuelos et al. 2009)
	Specific CB2R stimulation of rats stereotaxically treated with malonate (mitochondrial toxin)	CB2R stimulation protected neurons from malonate toxicity by inhibiting GABAergic neuron death and lowered striatal levels of proinflammatory cytokine TNFα	(Sagredo et al. 2009)
	Synthetic cannabinoid VCE-003.2 was tested against toxicity induced by (1) quinolinic acid–treated HiB5 progenitor cells, (2) mutant Htt-transfected STHdh cells, (3) quinolinic acid–treated mice, and (4) 3-NP-treated mice	VCE-003.2 treatment antagonized toxic effects by (1) enhancing cell viability and diminishing caspase 3 levels; (2) rescuing cell viability and limiting Htt aggregation; (3) limiting microgliosis and striatal neuron loss; and (4) improving motor deficits, inhibiting striatal neuron loss, lowering astroglial/microglial reactivity and prevented TNFα/IL-1β upregulation	(Díaz-Alonso et al. 2016)
ALS	rp-hTDP-43 (A315T) transgenic mice (exhibiting ALS traits) were treated with cannabinoid agonists such as HU-308	HU-308 inhibited motor neuron loss in the transgenic mice model, reduced the activation of GFAP-positive cells (astrocytes), diminished microglial reactivity, and improved the in vivo performance of mice on the rotarod test	(Espejo-Porras et al. 2019)
	SOD1^G93A^ transgenic mice were exposed to VCE-003.2 synthetic cannabinoid (PPARγ agonist)	VCE-003.2 treatment rescued impairments of the transgenic model by diminishing motor neuron loss and astroglial reactivity, while partially rescuing CB2R, IL-1β, GLT-1, and GLAST levels found in wild-type mice	(Rodríguez-Cueto et al. 2018)

## Nrf2 Signaling Pathway

### Overview

The nuclear factor erythroid 2-related factor 2 (Nrf2) is a basic leucine zipper (bZIP) transcription factor that, in humans, is structurally composed of seven Nrf2-ECH homology domains (Neh1-7). Neh1 provides Nrf2 with the bZIP-DNA binding domain, and Neh2 allows Nrf2 to interact with Kelch ECH-associating protein 1 (Keap1) (Canning et al. 2015).

Nrf2 function has been sometimes linked with inflammation (Ahmed et al. 2017), mitochondrial biogenesis (Hayashi et al. 2017), and autophagy (Pajares et al. 2018), as well as with amino acid, glucose, lipid, and purine metabolism (He et al. 2020). Nonetheless, Nrf2 is better known for its role in redox-homeostasis and ROS detoxification. Under basal conditions, Nrf2 is primarily localized within the cytosol, where it interacts with Keap1, which is able to recruit a specific E3 enzymatic complex. These interactions lead to Nrf2 poly-ubiquitinization and ultimately target Nrf2 for proteasomal degradation. In contrast, when ROS are overproduced, certain cysteine residues on Keap 1 become oxidized, preventing Keap1 from interacting with Nrf2 and inhibiting its proteasomal degradation (Ma 2013; Song et al. 2021).

Under oxidative stress conditions, Nrf2 translocates into the cell nucleus and binds to the antioxidant response element (ARE), a DNA sequence within the promoter region of Nrf2 target genes. In the nucleus, Nrf2 interacts with other transcriptional co-activators such as CREB-binding protein (CBP) and small masculoaponeurotic fibrosarcoma proteins (sMaf) (Katoh et al. 2001). Nrf2-ARE target genes are involved in a variety of cell metabolic pathways related to the antioxidant defense system, including (1) the thioredoxin (TRX)-antioxidant system, controlling the expression of TRX, sulfiredoxin (Srx), and thioredoxin reductase (TrxR) (Tanito et al. 2007; Tonelli et al. 2018); (2) the glutathione-antioxidant system, regulating the expression of glutathione peroxidase 2 (Gpx2), glutathione reductase 1 (Gsr1), and both subunits of gamma-glutamylcysteine synthetase (GCS), as well as several glutathione S-transferases (Chan and Kwong 2000; Chanas et al. 2002; Singh et al. 2006; Tonelli et al. 2018); (3) Nrf2 modulates nicotinamide adenine dinucleotide phosphate (NADPH) synthesis, and controls the expression of enzymes that detoxify ROS through NADPH consumption, such as NADPH-quinone dehydrogenase 1 (Nqo1) (Wu et al. 2011; Tonelli et al. 2018); (4) Nrf2 also upregulates the expression of phase I, phase II, and phase III xenobiotic-metabolism enzymes (Lubelska et al. 2016; Tonelli et al. 2018); (5) Nrf2 participates in iron metabolism, particularly upregulating the expression of ferritin, a protein that prevents iron from participating in ROS production via Fenton reaction; (6) finally, but not less important, key antioxidant genes involved in the regulation of redox homeostasis, including superoxide dismutase (SOD), catalase (CAT), and heme oxygenase-1 (HO-1), are also well-known targets for Nrf2 (Zhang et al. 2021a, b). Upregulation of all these proteins neutralizes the overproduction of ROS, thereby limiting oxidative damage and ultimately keeping cell homeostasis (Kerins and Ooi 2018).

### Physiological Significance in the Nervous System

Within the nervous system, the transcriptional activity of Nrf2 is involved in a considerable number of physiological processes. Firstly, Nrf2 appears to have a role in neurogenesis and neuronal differentiation as it has been reported that (1) mice lacking Nrf2 show impaired proliferation/differentiation of neural stem cells within the subgranular zone of the hippocampus (Robledinos-Antón et al. 2017); (2) Nrf2 upregulation promoted the migration, proliferation, and differentiation of neural stem cells of the subventricular zone of aged rats (Anandhan et al. 2021); (3) Nrf2 expression mediated the differentiation of neural stem cells into mature neurons or glia in the dentate gyrus of middle age rodents (Ray et al. 2018).

The Nrf2 signaling pathway also crosstalk with neurotrophin signaling in the nervous system. It has been proposed that brain-derived neurotrophic factor (BDNF) and nerve growth factor (NGF) (neurotrophins) might interact with p75 neurotrophin receptors to stabilize Nrf2 (Ishii et al. 2019). In addition, Nrf2 attenuates the expression of transcriptional repressors of BDNF, while directly enhancing its transcription (Yao et al. 2021). Furthermore, studies in human astrocytes showed that Nrf2 also promotes NGF expression (Mimura et al. 2011). Therefore, neurotrophins and Nrf2 form a positive feedback circuit that might enhance neuronal survival, as well as antioxidant defense-gene expression, in the nervous system.

Notably, Nrf2 is also thought to be involved in synaptic plasticity and memory formation. Recently, it has been reported that Nrf2 is able to enhance the expression of a circular RNA molecule that indirectly enhances CaMKIV expression, thereby promoting hippocampal plasticity and memory formation (Zhang et al. 2022). In agreement, allicin-dependent Nrf2 activation proved to partially rescue the cognitive dysfunction observed in aged mice (Li et al. 2012).

Additional studies reported several other functions of Nrf2. For instance, Nrf2 raised the expression of the mitochondrial biogenesis-related protein PPARγ-coactivator 1α (PGC1α) in the hippocampus, prefrontal cortex, and amygdala (Khalifeh et al. 2017). Several studies reported that Nrf2 activators enhance microglial polarization towards an M2 neuroprotective phenotype (He et al. 2021a, b; Tao et al. 2021; Wang et al. 2023a, b, c). Nrf2 is also activated in astrocytes to counteract Fe^2+^-induced toxicity (Cui et al. 2016).

### Role on the Physiopathology of Neurodegenerative Disorders

As noted above, Nrf2 impacts the physiology of the nervous system in many different manners; however, its primary function involves the direct modulation of redox-homeostasis, a function that is thought to be compromised in many NDs (Heurtaux et al. 2022). In AD *postmortem* brains, reduced nuclear translocation of Nrf2 was observed within the hippocampus and frontal cortex of AD tissues (Ramsey et al. 2007). In contrast, an efficient Nrf2 nuclear translocation has been reported in PD *postmortem* brains, although an inconsistent reduction of Nqo1 has been observed in other models, which could suggest that even if Nrf2 is being translocated, the expression of its target genes might remain altered in PD (Ramsey et al. 2007; Cook et al. 2011). Moreover, both PD and AD have been related with certain variants of *NFE2L2* (Nrf2 coding gene) that appear to correlate to the onset of these diseases (von Otter et al. 2010, 2014). In HD patient-derived neural stem cell cultures, inhibition of the Nrf2 signaling was noted (Quinti et al. 2017). Regarding ALS patients, reduced mRNA levels of Nrf2 were found in the spinal cord and motor cortex, while increased Keap1 levels were reported only in the motor cortex (Sarlette et al. 2008). Thus, even though Nrf2 signaling impairment varies in different NDs, the alterations of the overall pathway appear to be a constant factor.

Increasing evidence suggests that the enhancement of Nrf2 signaling can result in overall neuroprotection, as shown with a variety of in vivo and in vitro models linked to different NDs (Table 3). The evidence consistently suggests that Nrf2 activation results in neuroprotection, and some studies are even starting to consider the use of Nrf2 activators, such as sulforaphane, for clinical trials (Kim 2021).

**Table 3:** Main experimental results of studies reporting neuroprotective effects of Nrf2 signaling activation against NDs: AD, PD, HD, and ALS. *MDA* malondialdehyde; *HO-1* heme oxygenase 1; *PI3k* phosphatidylinositol 3-kinase; *Akt* protein kinase B; *SOD* superoxide dismutase; *LPS* lipopolysaccharide; *MPTP* 1-methyl-4-phenyl-1,2,3,6-tetrahydropyridine; *CSPQ* quercetin-modified ultrasmall Cu_2-x_Se nanoparticles; *NLRP3* NOD-, LRR-, and pyrin domain-containing protein 3; *Sirt1* Sirtuin 1

Disease	Experimental approach	Main results of Nrf2 signaling potentiation	Reference
AD	Indirect Nrf2 induction by resveratrol was tested on Aβ (1–42)-treated PC12 cell cultures	Resveratrol treatment rescued cell viability of cell cultures exposed to Aβ through the PI3k/Akt/Nrf2/HO-1 pathway by increasing GSH levels and decreasing ROS production/MDA levels	(Hui et al. 2018)
	Tg2576 AD transgenic mice were treated with 3H-1,2-dithiole-3-thione; an Nrf2 activator	3H-1,2-dithiole-3-thione induced the Sirt1/Nrf2 pathway potentiation and reduced insoluble-Aβ levels, while improving the cognitive decline of rodents, hippocampal neurogenesis, and expression of hippocampal GSH, SOD, and Gpx	(Cui et al. 2018)
	Different mice AD models (3x-Tg and 5xFAD), as well as different cell lines (MEF, SH-SY5Y, and HEK293T), were treated with sulforaphane, a well-known Nrf2 inducer	As proved with the cell culture models, Nrf2 binds to ARE sequences within the β-secretase 1 promoter region, thereby restricting its expression. In mice models, sulforaphane treatment reduced β-secretase 1 expression, diminished Aβ production, and ameliorated cognitive impairments in vivo	(Bahn et al. 2019)
	APP/PS1 transgenic AD mice and Aβ-exposed HT22 cells were treated with eriodictyol	Eriodictyol acted through the vitamin D receptor/Nrf2 pathway to inhibit ferroptosis, while reducing oxidative damage, Aβ aggregation, tau hyperphosphorylation, and oxidative damage in both models. Eriodictyol also improved cognitive deficits observed in APP/PS1 mice	(Li et al. 2022)
PD	The Nrf2 activator KKPA4026 was tested on (1) BV2 microglia-like cells, (2) LPS-activated BV2 cells, and (3) MPTP-treated mice	KKPA4026 induced (1) an increase of NQO1, HO-1, and both subunits of GCS; (2) a reduction of IL-1β, NO, iNOS, and TNFα levels; and (3) a reduction of dopaminergic neuronal loss and microglial activation, while improving the mice performance on the rotarod test, the coat-hanger test and the vertical grid test	(Kim et al. 2020)
	Different cell culture models (primary neurons and MES23-5 cells), as well as C57BL/6 mice treated with α-Syn fibrils, were used to test quercetin-modified ultrasmall Cu_2-x_Se nanoparticles (CSPQ)	CSPQ nanoparticles induced TFEB-dependent Nrf2 activation. Nrf2 enhanced SQSTM1/p62-dependent autophagy and reduced the aggregation of α-Syn in α-Syn-treated primary neurons. Besides, in α-Syn-treated mice, CSPQ reduced α-Syn levels and dopaminergic neuron loss and improved the mice performance on the Morris water maze and the rotarod test	(Liu et al. 2023)
	Hippocampal neurons were isolated and cultured from A53TSyn mice embryos. Primary cultures were tested against dimethyl fumarate treatment (Nrf2 activator)	Dimethyl fumarate treatment attenuated the deficits observed in the A53TSyn-mice neurons by improving mitochondrial respiration, dendritic arborization, and dendritic spine density, while reducing ROS production	(Brandes et al. 2021)
	The phytocompound gintonin (Nrf2 regulator) was injected into C57BL/6 mice previously treated with MPTP toxin	Through the Nrf2/HO-1 pathway, gintonin ameliorated proapoptotic/proinflammatory protein expression, α-Syn production, ROS/MDA generation, and astroglial/microglial activation. Gintonin also enhanced dopaminergic neural survival, Bcl2 expression, and in vivo motor performance	(Jo et al. 2019)
	Celastrol treatment was injected into (1) MPTP-exposed mice; and (2) AVV-induced human α-Syn-expressing mice	Celastrol acted through Nrf2 activation, which in turn inhibited NLRP3/caspase1-dependent inflammation and cell death. Celastrol diminished neuronal loss and motor deficits in both experimental models	(Zhang et al. 2021a, b)
HD	Wistar rats were treated with diapomycin prior to a treatment with 3-NP striatal neurotoxin	Diapomycin treatment protected rats from 3-NP-induced damage through Sirt1/Nrf2 pathway by inhibiting glial reactivity, deactivating proapoptotic pathways, reducing inflammatory/oxidative markers, and improving rat-motor function	(Ibrahim and Abdel Rasheed 2022)
	Several luteolin derivatives were tested on mutant hunting-expressing STHdh-cells	Luteolin and derivatives proved to reduce caspase-3 levels and ROS production. Particularly, two derivatives showed an enhancement of Nrf2, and one of them increased the activity and mRNA levels of SOD and one of the subunits of GCS	(Oliveira et al. 2015)
ALS	SOD1^G93A^ transgenic mice were injected with tetramethylpyrazine nitrone, an inducer of Nrf2 activity PGC1α/Nrf2/HO-1 pathway	Nrf2 activation diminished transgenic SOD expression, muscular atrophy/fibrosis, astroglial/microglial activation, MDA levels, and motor neuron loss. Nrf2 potentiation also improved the in vivo performance on the grip strength test, climbing time in pole test, and hanging time in wire test	(Wen et al. 2021)
	S[ +]Apomorphine (Nrf2 activator) was tested on different ALS models	S[ +]Apomorphine improved motor function and muscle innervation in SOD1^G93A^ mice while decreased ROS production in fibroblasts isolated from ALS patients	(Mead et al. 2013)

## Proteasome ↔ Nrf2 Interactions

Both UPS and Nrf2 signaling are interconnected and interfere with the regulation mechanisms of each other. Firstly, Nrf2 enhances the expression of several proteasome-subunits and increases overall proteasomal activity (Kwak et al. 2003; Yang et al. 2007; Arlt et al. 2009; Malhotra et al. 2009; Kapeta et al. 2010; Tsakiri et al. 2013). In corroboration, numerous experimental models have suggested that Nrf2 exerts neuroprotection through proteasome activity improvement. For instance, (1) in Neuro 2A neuroblastoma cells, sulforaphane (Nrf2 activator)-induced neuroprotection against Aβ-induced cytotoxicity was reported to involve the proteasome activity, as proteasome inhibitor MG132 reduced the cytoprotection achieved (Park et al. 2009); (2) a toxic model of adenoviral vector-induced α-Syn-expression in Nrf2-lacking mice showed that Nrf2 deficiency downregulated the expression of several proteasome-subunits, contributing to α-Syn-aggregation, glial reactivity, and overall dopaminergic neuron damage (Lastres-Becker et al. 2012); (3) in SK-N-BE neuroblastoma cell line, 24-hydroxycholesterol treatment indirectly induced Nrf2 activation, consequently activating the proteasome and enhancing Tau protein clearance, an effect that was antagonized by Nrf2-silencing (Testa et al. 2023); (4) in two different α-Syn-overexpression PD models, it was reported that the upregulation of Nrf2/proteasome maturation protein (POMP) pathway enhanced immunoproteasome assembly, thereby leading to α-Syn degradation (Bi et al. 2021); (5) sulforaphane treatment, a well-known Nrf2 enhancer, has shown to protect N2a neuroblastoma-cell cultures from hydrogen peroxide toxicity. This cytoprotection proved to be UPS-dependent, as proteasome inhibition markedly reduced the neuroprotective effects of sulforaphane, which increased the mRNA levels of a number of Nrf2 target genes, including CAT, Nqo1 and the regulatory subunit of GCS (Kwak et al. 2007).

In a heart failure model, Nrf2 enhancement upregulated proteasomal activity, reducing the accumulation of damaged proteins and resulting in overall cardioprotection. In addition, in an insulinoma β-cell line, Nrf2 cytoprotection against tunicamycin was achieved throughout proteasome activity potentiation (Lee et al. 2012; Shimizu et al. 2016). Notwithstanding the above, other studies in different cell lines suggested that DJ-1 protein-mediated Nrf2 activation could both activate and inhibit the proteasome through different pathways (Moscovitz et al. 2015).

As previously reviewed, the UPS also regulates Nrf2 stability and degradation (Ma 2013; Song et al. 2021) and conversely to the previous idea, most evidence suggests that proteasomal inhibition could enhance Nrf2 signaling and consequently achieve neuroprotection. For instance, (1) in 6-hydroxydopamine (6-OHDA)-treated PC12 cells, proteasome inhibitor lactacystin has been shown to reduce oxidative stress, enhanced Nrf2 translocation/Nrf2 target gene expression, and upregulated glutathione levels (Yamamoto et al. 2007; Izumi 2013), (3) paraquat treatment toxicity was attenuated by lactacystin and MG132 (proteasome inhibitors), and consistently, both inhibitors increased the transcriptional activity of Nrf2 (Izumi et al. 2015). Nevertheless, fewer studies have contradictory conclusions, as a genetic-induced inhibition of the 26S proteasome proved to diminish Nrf2, Nqo1, and heme oxygenase 1 (HO-1) protein levels (Ugun-Klusek et al. 2017).

Therefore, whether proteasomal inhibition enhances or decreases Nrf2 signaling remains unclear. However, given that UPS impairment appears to be a pathological hallmark of multiple NDs (Thibaudeau et al. 2018), therapeutic inhibition of the proteasome might not be the best approach to reach Nrf2-signaling potentiation, considering that Nrf2 stabilization can be achieved with absent proteasomal inhibitors, as proven with *tert*-butylhydroquinone in human neural stem cells (Li et al. 2005).

## Nrf2 ↔ Endocannabinoid System Interactions

As reviewed in previous sections, Nrf2/ECS potentiation has been considered a promising therapeutic approach against NDs; however, minor attention has been focused on the interaction between these two major signaling pathways. Increasing evidence suggests that several ECS-related receptors such as CB1R, CB2R, and TRPV1 regulate Nrf2 activity as shown with different tissues and cell models (Li et al. 2016; Lv et al. 2021; Baradaran Rahimi and Askari 2022; Mageed et al. 2022).

Many studies have used cannabinoid signaling as an indirect pathway to achieve Nrf2-dependent neuroprotection, for example, (1) primary neuron cultures were exposed to a toxic treatment of Aβ-oligomers and high glucose; afterwards, the cells were tested with different cannabinoid-related agents including endogenous/synthetic agonists as well as a FAAH inhibitor. All agents upregulated Nrf2, and differentially protected cells by increasing their viability, while reducing Aβ levels and inflammation/oxidative damage-related markers (Elmazoglu et al. 2020); (2) TRPV1 inhibition by a peptide-antagonist resulted in Nrf2 activation, reduction of Aβ processing, accumulation and Aβ precursor protein levels in amyloid precursor protein-over expressing N2a cells (Wang et al. 2022a, b, c); (3) in turn, SOD, CAT, GPx, and Gsr might be regulated by cannabinoids (Elmazoglu et al. 2020), hence highlighting the interaction between these systems.

Further evidence is based on ischemic damage models on neurons, which share some cell damage markers (e.g., oxidative stress and apoptosis) with NDs. For instance, MAGL inhibition rescued oxygen-deprived hippocampal neurons by diminishing apoptosis, as well as oxidative and pro-inflammatory markers, while improving Nrf2 signaling and cell viability. Both the changes observed in cell viability and oxidative markers were partially blocked when silencing Nrf2, thereby demonstrating that cannabinoid upregulation was acting through the Nrf2 pathway to block oxygen deprivation effects (Xu et al. 2021),

Although Nrf2 activation through cannabinoid signaling potentiation has been observed in many cell types, it appears to be particularly important for microglia (Li et al. 2013; Tadijan et al. 2022; Wang et al. 2022a, b, c). Microglia is known to express CB2R receptors and CB2R activation is thought to regulate microglial reactivity, therefore preventing inflammatory damage in several models of NDs (Ashton and Glass 2007; Komorowska-Müller and Schmöle 2020; Young and Denovan-Wright 2022). Outstandingly, it appears that this process might be regulated via Nrf2 signaling, as CB2R agonist JWH133 proved to prevent 1-methyl-4-phenylpyridinium (MPP +)-stimulated microglia from differentiating into a M1 proinflammatory/neurotoxic phenotype, while enhancing their differentiation into a M2 anti-inflammatory/neuroprotective phenotype, an effect that was reverted by Nrf2 inhibition (Wang et al. 2023a, b, c). Additionally, a study in macrophages found similar results, as TRPV1-dependent ion fluxes resulted in an increased Nrf2 signaling and consequently a decreased macrophage differentiation into a M1 phenotype (Lv et al. 2021).

Further evidence based on BV2 microglia-like cells suggested that phytocannabinoids such as cannabidiol (CBD) and Δ9-tetrahydrocannabinol (Δ9-THC) inhibit NF-kB and interferon β/Signal transducer and activator of transcription (STAT) proinflammatory signaling pathways, which might complement the concept that Nrf2 is indirectly able to inhibit NF-kB (Kozela et al. 2010; Wardyn et al. 2015). This previous study also reported that Δ9-THC and CBD act independently from CB1R/CB2R receptors, suggesting that cannabinoids might achieve neuroprotection in this regard through different mechanisms (Kozela et al. 2010). In addition, a microarray analysis of lipopolysaccharide-stimulated BV2 cells showed that mainly CBD is able to upregulate Nrf2, which supports the previous argument (Juknat et al. 2013).

To our current knowledge, little evidence has been found regarding the Nrf2-dependent regulation of the ECS components. In 2020, it was found an ARE sequence within the promoter region of the CB2R-coding gene, where Nrf2 was only reported to regulate CB2R expression in microglia (Galán-Ganga et al. 2020). This report opens up the debate of whether Nrf2 upregulation could enhance cannabinoid signaling via CB2R, boosting the neuroprotective effects of this cannabinoid receptor regarding microglial reactivity and polarization, as previously mentioned.

Finally, cannabinoid-responsive PPARγ receptors are thought to enhance Nrf2 expression as PPARγ response elements in the DNA have been found in the promoter region of *NFE2L2*. On the other hand, PPARγ is also upregulated by Nrf2, thereby shaping a positive feedback loop between these two transcription factors (Cho et al. 2010; Huang et al. 2010; Lee 2017; Lin et al. 2018).

Evidence regarding PPARγ/Nrf2 interactions is predominantly derived from ischemic/hemorrhagic stroke studies. For instance, (1) PPARγ-agonist pioglitazone was tested in different models of middle cerebral artery occlusion and reperfusion (MCAO); PPARγ activation reduced apoptosis while improving neurological score of rats in vivo, besides it alleviated oxidative damage/excitotoxicity in 6-OHDA/glutamate-treated cells, respectively (Zhao et al. 2021), (2) MCAO-treated rats were exposed to luteoloside, a compound that proved to increase PPARγ and Nrf2 expression, possibly leading to the inhibition of nuclear factor-kB (NF-kB) and the expression of its inflammation-related target genes (Li et al. 2019a, b), (3) sirtuin 1 (Sirt1) protein also proved to exert neuroprotective effects in an ischemia model through the PGC1α/PPARγ/Nrf2 pathway to reduce oxidative stress and enhance cell viability (Zhou et al. 2022). All these models show that ND-related markers such as oxidative damage, neuroinflammation, and excitotoxicity can decrease through the PPARγ/Nrf2 pathway, potentially via PPARγ agonists such as cannabinoid-like compounds.

Ferroptosis is an iron-dependent cell death pathway that has been related to AD, PD, and HD pathologies (Tang et al. 2021); however, the existing evidence is again based on hemorrhagic stroke studies. In intracerebral hemorrhage models, pioglitazone was used to induce the PPARγ/Nrf2 axis, consequently inhibiting ferroptosis in erastin-treated neuron cultures and rats with an intracerebral blood injection (Duan et al. 2022). Additionally, in subarachnoid hemorrhage models, it was proven that the activation of the PPARγ/Nrf2 axis via netrin-1 reduced ferroptotic cell death through the enhancement of Gpx4 and coenzyme-Q10/ferroptosis suppressor protein-1 pathways (Chen et al. 2023). Neuroprotective effects of the PPARγ/Nrf2/Gpx4 pathway against ferroptosis have also been described in an epilepsy model tested on rats (Wang et al. 2023a, b, c). Taken together, these results suggest that the PPARγ/Nrf2 axis might aid in targeting ferroptosis in several NDs.

In addition to stroke models, PPARγ signaling has been tested in inflammation-related studies. NLRP3-inflammasome activation has been shown to be toxic in AD, PD, HD, and ALS models, and several of them suggest that NLRP3 inhibition could exert neuroprotective effects in these NDs (Deora et al. 2020; Hanslik and Ulland 2020; Haque et al. 2020; Chen et al. 2022). Indirect evidence from an alcoholic liver damage model has found that phyto-derivative magnolol is able to increase the levels of PPARγ and Nrf2, while reducing NLRP3 proinflammatory-signaling cascade and the damage associated with it (Liu et al. 2019). In corroboration, both PPARγ and Nrf2 are reportedly able to inhibit NLRP3 (Hou et al. 2018; Yang et al. 2021). Although to our current knowledge, it is still unproved, the PPARγ/Nrf2 axis could reduce ND-related neuroinflammation/cell death through the inhibition of NLRP3.

Finally, a PD-related neuroinflammation model induced by lipopolysaccharide demonstrated that a pioglitazone treatment could improve the behavioral impairment in vivo, while increasing dopaminergic neuron survival within the SN through the activation of the Nrf2/HO-1 pathway and the inhibition of NF-kB signaling (Zakaria et al. 2019).

## Proteasome ↔ Endocannabinoid System Interactions

In contrast to the interactions reviewed in previous sections, limited evidence supports a regulatory linkage between the ECS and the UPS pathways. Regarding these interactions, it has been proposed that the phytocannabinoid Δ9-THC is able to inhibit the proteolytic activity of the 20S core particle in the hippocampus (Salgado-Mendialdúa et al. 2018). Additionally, further studies on human astrocytes suggested that Δ9-THC modulated the expression of several genes related to the ubiquitin pathway (Bindukumar et al. 2008). However, the available evidence remains controversial.

There are two main reports that suggest a possible cooperation between the UPS and the ECS to achieve neuroprotection in ND-related models. The first one reported that in N2a cells transfected with mutant Htt, activation of the cannabinoid-related receptor PPARγ by the agonist rosiglitazone rescued cell viability and diminished several markers of cell damage; however, rosiglitazone also enhanced the proteolytic activity of the proteasome. This result is supported by the fact that rosiglitazone also reduced the aggregation of mutant Htt and that one of the PPARγ target genes is part of the proteasome activator complex (Chiang et al. 2015). The second report concluded that in *C. elegans* nematodes of different strains, CBD treatment increased dopaminergic neuron survival and the lifespan of the worms, while reducing oxidative damage markers. In the strain OW13 (that expresses α-Syn in muscular cells), CBD treatment proved to increase the activity of the proteasome and consistently decreased α-Syn accumulation (Muhammad et al. 2022). These results suggest that cannabinoid potentiation might enhance the UPS activity, potentially exerting neuroprotection against proteostasis impairments.

It is noteworthy that ECS potentiation has been linked with proteasomal UPS activity in other studies less related to NDs. (1) It has been reported that genetic inactivation of CB1R alters myelination and disrupts the differentiation of oligodendrocytes, leading to motor and cognitive deficiencies in vivo, and additionally, it was found that CB1R promoted oligodendrocyte-precursor cell differentiation via the enhancement of the proteasome-dependent degradation of Ras homolog family member A. These previous observations offer a potential therapeutic approach in experimental models of ALS, where myelination and oligodendrocyte differentiation appear to be disrupted (Raffaele et al. 2021; Sánchez-de la Torre et al. 2022), (2) CB1R receptor activation was also linked with neurite outgrowth in N2a cells, a process that is thought to involve the targeting of Rap1-GTPase activating protein II to ubiquitination and proteasome-dependent degradation. These results are of great relevance, given that neurotoxic proteins such as Aβ and α-Syn are able to inhibit neurite outgrowth, a phenomenon that might be targeted through the CB1R/proteasome pathway (Takenouchi et al. 2001; Jordan et al. 2005; Calkins and Reddy et al. 2011).

## Concluding Remarks

**Fig. 1:**
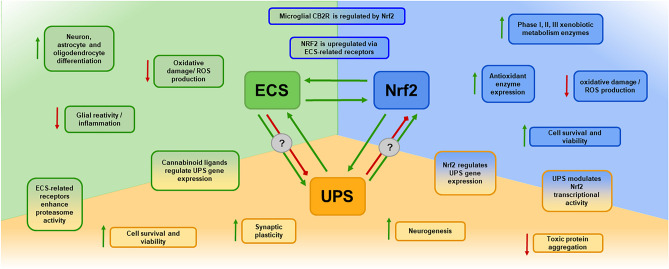
Summary of the reported interactions and effects between ECS, UPS, and Nrf2 systems. Green arrows represent stimulatory effects, while red arrows represent inhibitory effects

In summary, as NDs are multifactorial, this review provides evidence in support of the involvement of UPS, ECS, and Nrf2 signaling impairment in the origin and progression of several NDs. Experimental manipulation of the UPS, ECS, and Nrf2 has shown promising results as potential therapeutic approaches for NDs. However, particular attention should be focused on the interactions between these pathways, as evidence shows that synergic neuroprotective effects might be achieved when enhancing the activity of Nrf2, ECS, or UPS. Despite the potential of therapeutically targeting these in NDs, the available data are limited as the available evidence is not directly derived from NDs models but rather from ischemic neuronal damage or other non-neuronal cytoprotection models; therefore, further research will be needed to decipher the actual neuroprotective efficacies of the synergic enhancement of Nrf2, ECS, or UPS. A graphical representation summarizing the interaction of the Nrf2, ECS, and UPS systems is shown in Fig. 1.

Finally, by recommendation of one reviewer of the manuscript, we include this following section with suggested reviews on the overviews developed herein that may be of support in certain aspects. https://www.sciencedirect.com/science/article/pii/S2213231722002610. https://www.sciencedirect.com/science/article/pii/S0753332223016037. https://www.mdpi.com/2076-3921/12/7/1371. https://www.mdpi.com/1422-0067/24/13/11003. https://www.ncbi.nlm.nih.gov/pmc/articles/PMC9710321/. https://www.annualreviews.org/doi/full/10.1146/annurev-pharmtox-052220-103416. https://molecularneurodegeneration.biomedcentral.com/articles/10.1186/s13024-015-0012-0.

## Data Availability

Not applicable
